# The Effect of a Novel Video Game on Young Soccer Players' Sports Performance and Attention: Randomized Controlled Trial

**DOI:** 10.2196/52275

**Published:** 2024-05-27

**Authors:** Adrian Feria-Madueño, Germán Monterrubio-Fernández, Jesus Mateo Cortes, Angel Carnero-Diaz

**Affiliations:** 1 Department of Physical Education and Sport, University of Seville, Sevilla, Spain; 2 Sport Department, Centro de Estudios Universitarios San Pablo Cardenal Spinola University, Bormujos, Spain; 3 Fitness and Rehab Coach, Hammarby Fotboll AB, Arsta, Sweden

**Keywords:** reaction time, serious games, executive function, decision making, game, games, gaming, sport, sports, soccer, football, athlete, athletes, athletic, training, performance, physiological, muscle, muscular, sweat, sweating, attention training, attentional, ball, exercise, physical activity, exergame, exergames, interview, interviews

## Abstract

**Background:**

Currently, the fusion of technology and sports is inevitable. The integration of various systems and devices has brought about significant transformations in established sports practices, impacting not only the rules but also physiological, biomechanical, and even psychological aspects.

**Objective:**

The purpose of this study was to analyze the effect of an attention intervention through a video game on young soccer players.

**Methods:**

Twelve young male soccer players (age: mean 8.5, SD 1 years) were divided into 2 groups: a control group (CG; n=10) and an experimental group (EG; n=10). During the 6-week training program, the EG received attention training through a video game twice a week for 15 minutes per session. Pre- and postintervention measurements included a specific decision-making soccer test and interviews with coaching staff. Additionally, success in the video game, muscular activity, and sweat levels were monitored.

**Results:**

The EG demonstrated a significant improvement in video game success following the intervention program, as indicated by the achieved level (*P*<.001). However, no significant differences were found between groups regarding electromyographic (EMG) activity (*P*=.21) and sweating (*P*=.20). Prior to implementing the attention training program, both groups exhibited similar data for variables related to decision-making and execution mechanisms (≤10%). Only 2 decision-making variables exceeded 10% but remained below 15% (Shot_D=13.35%; Marking_with_Ball_D=−12.64%). Furthermore, changes in attacking action variables were more pronounced in execution-related variables, except for dribbling and fixing. Conversely, in defensive action variables, changes were greater in decision-related variables, except for marking with the ball and marking without the ball.

**Conclusions:**

Our findings reveal that incorporating a specific attentional video game into a soccer training program enhances decision-making compared to a program without the video game. Therefore, it is advisable for practitioners to consider using this tool due to its high efficiency in terms of economic and temporal costs, particularly in improving a key psychological variable.

## Introduction

Currently, technology use is inseparable from sports. The integration of various systems and devices has brought about significant transformations in established sports practices, impacting not only the rules but also physiological, biomechanical, and even psychological aspects. This revolution is particularly evident in soccer, where the implementation of technology has primarily concentrated on the professional sphere. From a physiological perspective, technology has facilitated notable advancements in the understanding and control of physical demands during elite soccer matches. Tools such as the GPS [1,2], accelerometry, and specialized match analysis programs [2] have made it possible for coaches to design training tasks on the basis of data obtained from actual games. Optical tracking systems and GPS devices were compared in professional soccer, revealing no significant differences in analyzed variables such as total distance, distance per minute, average speed, and maximum speed [3]. However, limitations were identified, such as the ineffectiveness of the GPS in indoor sports and the inability of optical tracking systems to access internal variables. Nevertheless, researchers concluded that both technologies were suitable for monitoring the physiological demands of soccer players [3].

Technological applications in soccer have also shed light on performance mechanisms in areas such as jumping [4] and the minimization of lower limb injuries [5]. Usually, these technologies rely on 3D movement analysis, muscle activation assessment, and force production evaluation. Nonetheless, the high economic cost and specialized expertise required for their use pose significant challenges, making it difficult to implement them in the daily lives of athletes. Another way to harness the use of technology with a better cost-benefit relationship is through the use of video games; in particular, those with a clear aim to improve performance and learning, known as serious games [6]. Games have been used to introduce challenging concepts or develop skills in different areas such as surgery [7] and rehabilitation [8] or to enhance team coordination via cooperative training—the latter being called “small side games” [9,10]. All manner of play seeks to achieve improvements in skill acquisition in a more attractive way over and above traditional forms [11,12]; in particular, among pediatric populations with special attention and motivation characteristics [13,14]. Playful forms of intervention have improved affective responses in children compared to traditional teaching methodologies [15], having achieved benefits with the use of video games that optimize performance in variables as attention and executive control [16] and other psychological variables [17]. Serious games are emerging as valuable tools with positive impacts on cognitive aspects, providing specific value to their users. A pertinent study demonstrated that the implementation of a serious game based on chess, titled “The Secret Trail of Moon,” led to notable increases in emotional control levels and a reduction in attention deficits among participants [18]. In the sports domain, literature also highlights benefits, with a serious virtual reality game showing a significant improvement in players’ concentration during skiing tasks [13]. These findings underscore the potential positive impact of serious games on diverse cognitive and sports-related domains. Studies have increasingly focused on the psychological and cognitive aspects of athletes over the years [19,20], having highlighted attention, memory, and motivation as crucial psychological variables for athletes [21,22]. While the relationship among technology, games, and the psychological behavior of athletes has been explored, research specifically focusing on technology training in soccer remains scarce compared with other areas of knowledge. A notable study [23] revealed that young soccer players who trained with a computerized attention training system called Rejilla (version 1.0) [24] experienced significant improvements in their attention. However, these improvements were not directly linked to on-field performance variables. The literature has primarily centered around elite soccer, with limited attention given to youth soccer. Providing information to youth soccer coaches regarding how technology can impact the psychological and cognitive variables of their athletes will help optimize performance and achieve long-term success.

## Methods

### Participants

The study involved 12 young male soccer players aged between 8 and 9 years from a club in Seville, Spain. The participants underwent training sessions 3 days a week, each lasting approximately 120 minutes. The sample was divided into 2 groups: a control group (CG) and an experimental group (EG). Both groups were informed about their participation in a program aimed at enhancing their soccer skills. For the CG, the training involved watching videos of goals scored by the first team in previous seasons, followed by researchers posing questions to elicit responses. In search of a placebo effect, the EG was informed that the activity they were engaging in was attention training. This involved the visualization of videos showcasing goals scored by the first team in past seasons. After the video session, participants were required to answer questions related to the content (eg, “how many goals did the player with the number 9 score?”). Alternatively, the EG engaged in attention training using a video game.

Sample size calculation was convenience-based. For this, the study participants were players from a youth team belonging to a top-tier club in the Spanish Professional Football League. As an inclusion criterion, participants had to be 8 or 9 years old. Additionally, they should have been free from injuries that would have prevented them from participating in training or competitions. Their required training frequency was 3 times per week. During the 6 weeks, if any player reduced their training frequency to once a week, they were withdrawn from the study. Finally, a simple randomization method was chosen to randomize the groups, using the toss of a coin.

### Procedure

**Figure 1.**
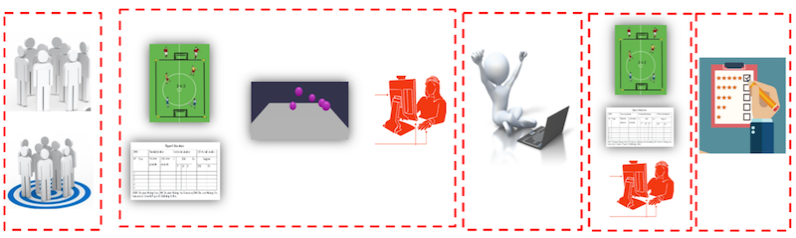
Study design. Left to right: participants were randomized into 2 groups. Subsequently, all soccer players were assessed using the Game Performance Evaluation Tool (GPET), in addition to being evaluated for success in the video game, electromyographic (EMG) activity, and sweating levels. Afterward, the intervention proceeded for 6 weeks. Thereafter, both groups were evaluated again with the GPET, EMG activity, and sweating levels. Finally, the coaching staff was interviewed.

The study spanned a duration of 6 weeks (Figure 1). Initially, all players underwent an evaluation of their decision-making skills in relation to soccer performance using the Game Performance Evaluation Tool (GPET) test [25]. The GPET is a tool to assess performance in invasion sports, specifically in soccer. Subsequently, both groups were assessed on the basis of their success in the video game, electromyographic (EMG) activity during the test, and sweat level. Throughout the study, all participants maintained their regular training routine, consisting of 3 sessions per week and a competitive game. The intervention varied, in that, on the one hand, the CG attended a room session twice a week where videos showcasing goals scored by the first team in past seasons were shown during 15 minutes, and after each video, the investigators posed a question to elicit a response; on the other hand, the EG attended a room session where attention training was conducted using a video game, with sessions lasting 15 minutes and taking place twice a week. Upon completion of the 6-week intervention, all players were reassessed regarding their decision-making abilities in relation to soccer performance using the GPET, as well as their performance in the video game, EMG activity, and sweat level during video game practice. Finally, an interview was conducted with the coaching staff to gauge the subjective attention levels of each soccer player, assigning them a score ranging from 1 (very low attentional level) to 3 (optimal attentional level) in relation to competitive situations.

### Instruments

#### The Video Game: BallApp

The video game used in the study involved the task of memorizing a ball with a distinct color among others displayed on the screen. After a period of 5 seconds, all the balls would change to the same color and move randomly across the screen. The participants were then required to identify the ball that initially had a different color. Accomplishments in the video game were measured in both groups, with the criterion being the ability to advance to the next level 3 consecutive times to mitigate the influence of luck. As the levels progressed, the speed of the balls, the number of selectable balls, and the presence of distractors such as noise increased. To replicate the environment of competitive soccer matches, specific background noise recordings from the soccer players’ actual matches were provided.

The attention game for soccer players, BallApp, is designed to enhance their attention processes, aiming for a positive impact on attentional mechanisms when playing soccer. The game has been developed as an application using various technologies, including HTML for structural aspects, JavaScript for functionality, and Material Design for Bootstrap for design. The game is tailored for young soccer players and has been designed for individual play, although multiple players can participate on multiple screens.

The game involves selecting a ball of a different color than the others, and after a few seconds, all the balls turn into the same color and move randomly across the screen. The speed of movement, number of balls, depth of movement, and added noise vary as the difficulty levels progress. For example, in level 1, three balls appeared (one of them being of a different color), and the task was to choose 1 ball. All the balls moved without depth, and the speed was low. As levels increased, the difficulty level also increased on the basis of the number of balls on the screen. In subsequent levels, such as level 3, four balls appeared (one of them being of a different color), and the task was to choose 1 ball. The balls moved without depth, at a low speed, and without added noise. In total, the maximum number of programmed levels in the game was 8.

Level 4 onward, an ambient sound replicating those in real-world soccer matches was introduced. The aggressiveness of the noise increased with an increase in the difficulty level (Table 1). The aggressiveness of the noise was described on the basis of whether it was a murmur (low noise), cheering noise (medium noise), cheering noise with chants (moderate noise), noise with chants where specific phrases from a fan stood out (aggressive noise), or noise with chants and synchronized disturbances in the form of complaints or disgust toward plays (very aggressive noise).

**Table 1.** Overview of the characteristics of each level of the game.

Level	Number of balls on the display	Number of eligible balls	Depth of the movement	Speed of the balls	Sound
1	3	1	No depth	Low	Noiseless
2	3	1	With depth	Low	Noiseless
3	4	1	No depth	Low	Noiseless
4	5	1	No depth	Moderate	Slight noise
5	6	2	No depth	Moderate	Medium noise
6	6	2	With depth	Moderate	Moderate noise
7	7	2	No depth	High	Loud noise
8	8	2	With depth	High	Very loud noise

Regardless, to progress to a higher level, the player had to win the played level 3 times consecutively, eliminating luck as a factor for level progression. If the player correctly identified the balls of a different color in each level, they were awarded a score. This score depended on not only accuracy but also the time taken to make their choice.

The game has a configuration for individual use, although competitions can also be held with other players. Thus, it provides players with a leaderboard that compares scores among players, and this ranking changes on the basis of the following variables: the number of games played, the number of points achieved per game, and the number of levels surpassed by the player.

The selection of this particular video game for implementation is predicated on its consistent engagement of the attentional mechanism. This mechanism holds substantial implications in the developmental processes of young soccer players and, consequently, may yield positive effects on targeted soccer-related tasks.

#### EMG and Skin Conductance

**Figure 2.**
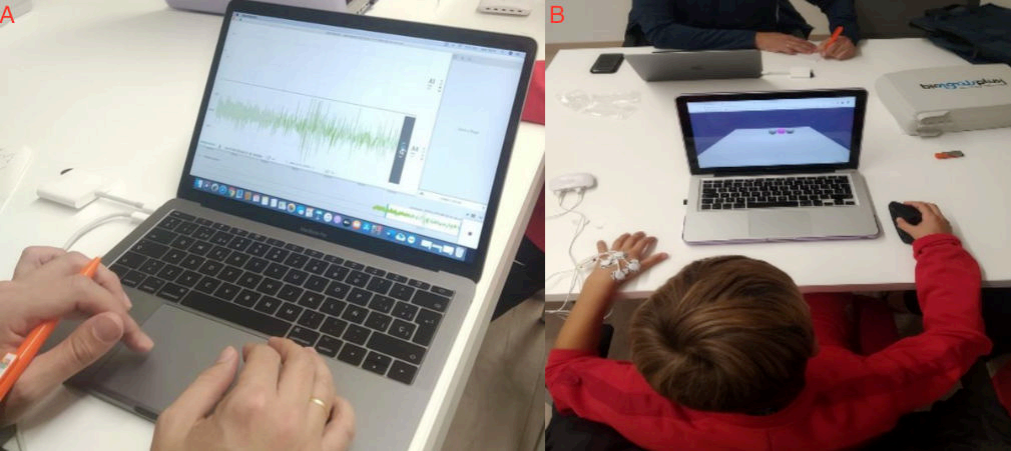
Evaluation of electromyographic activity (**A**) and electrodermal activity (**B**) during video game practice.

EMG activity and electrodermal activity (EDA) were assessed on the basis of skin resistance and sweat production (bioPLUX). The EMG activity and EDA of the dominant hand’s extensor muscle were evaluated in all participants. Electrodes were applied to the dominant hand, and these variables were analyzed before and after the intervention program during video game practice. These variables were examined from a somatic perspective, allowing for the monitoring of psychophysiological indicators during an attention-demanding task such as playing a video game (Figure 2).

The assessment of somatic variables of anxiety using biofeedback devices appears to be an excellent tool for controlling anxiety during different tests and among different populations [26], specifically in the analysis of EMG activity [27] and EDA variables [28], as specific control of somatic responses seems to be a good indicator of psychological states. Alterations in these variables could indicate somatic states and anxiogenic responses that would hinder the individuals’ task performance [29]. Our study monitored these 2 responses while participants played the video game.

#### GPET

The GPET assessment tool was used to evaluate decision-making in relation to soccer performance. This instrument differentiates the cognitive-decisional aspect of performance from execution. The playing field has dimensions of 20×10 m^2^, with delimited areas of 3×4 m^2^ and goals measuring 0.95×0.70 m^2^. The ball used is an A-7 soccer ball, with a circumference ranging between 0.635 and 0.66 m. The players engage in the task in teams consisting of 2 players each, with two 4-minute halves and a 3-minute break between them. The timer remains active throughout, and there are assistants designated to retrieve the ball. The variables captured by the test are assessed in both attacking and defensive situations, both with and without the ball (as outlined in Table 2). The test is recorded using a video camera for subsequent analysis. Using an observation sheet, successful actions are coded on the basis of the predefined roles into 4 options: appropriate decision (coded as 1), inappropriate decision (coded as 2), successful execution (coded as 1), and unsuccessful execution (coded as 2).

**Table 2.** Description of variables evaluated using the Game Performance Evaluation Tool.

	With the ball	Without the ball
Attack	Control Pass Dribbling Shot	Losing one’s defender Fixing
Defense	Marking with the ball Defensive blocking Tackle Clearing with the ball	Marking without the ball Interception Clearing without the ball

### Outcome Measures

All variables evaluated using the GPET are described below and are classified in accordance with two criteria (Table 2): (1) whether the action is carried out with or without the ball and (2) if the actions are those wherein the decisional or execution mechanism predominates. For this purpose, all acronyms used have been “_with_ball” for variables with the ball, “_without_ball” for variables without the ball, “_D” for variables where the decisional mechanism predominates, and “_E” where the execution mechanism predominates. Thus, the variables are the following: (1) control: this refers to situations where the player keeps the ball under control; (2) pass: this is the action where the player successfully makes a pass to another teammate; (3) dribbling: this is an action where the player gets away from his opponent, eliminating any possibility of the ball being taken away from him; (4) shot: the player takes a shot at goal with the aim of scoring; (5) Losing_one’s_defender: the player, by means of an attacking action, unmasks themself from their defender; (6) fixing: the player, in an attacking action, fixes the defender, forcing the latter to occupy a specific position on the field; (7) Marking_with_ball: in a defensive action, the player marks the player who has the ball; (8) Defensive_blockin: in a defensive action, the player tackles the player with the ball but does not fall to the floor; (9) tackle: in a defensive action, the player falls to the ground to snatch the ball from the opponent; (10) Clearing_with_Ball: in a defensive action, the player clears the player with the ball, using a free space; (11) Making_without_ball: in a defensive action, the player marks another player of the opposing team who is not in possession of the ball; (12) interception: in a defensive action, the player cuts off a play, preventing the ball from reaching its receiver; and (13) Clearing_without_ball: in a defensive action, the player makes a clearance to the player who does not have the ball, using a free space.

### Interview

Traditionally, the coach’s role as a key element in understanding the athlete has proven to be crucial [30]. Specifically, the athletes’ psychological factors have been tested using various procedures, with the coach playing a pivotal role as a key to understanding the athlete’s psychological status [31,32]. Thus, the qualitative insights obtained from interviews with coaches regarding the psychological variables of athletes can be a crucial element that significantly enhances the understanding of these psychological variables, aiding in decision-making about athletes [33].

An individual interview was conducted with the coach to subjectively assess the level of attention concerning decision-making and athletic performance for each soccer player. The coach was asked to rate, using a scale from 1 to 10, the level of attention exhibited by the soccer player in making optimal decisions that contribute to enhanced athletic performance. The coach provided a numerical rating, which was duly recorded by the evaluation team.

### Statistical Analysis

A statistical analysis was conducted on the data obtained for each variable. Initially, a parametricity analysis was performed for each variable using the Shapiro-Wilk test. Subsequently, the mean and SD of each variable were assessed. To compare the means between groups, an independent samples *t* test was used, with a significance level set at 95% confidence (*P*≤.05). Moreover, the effect size for the variables was determined using a 95% confidence limit. To qualitatively evaluate the potential quantitative changes observed after the program, the following categories were used [34]: highly improbable (<1%), very unlikely (1%-5%), unlikely (5%-25%), possible (25%-75%), likely (75%-95%), highly likely (95%-99%), and virtually certain (>99%).

### Ethical Considerations

This study received ethical approval from the Ethics Committee of the Center for University Studies affiliated with the University of Seville (20190215) on February 5, 2019. This study complied with the ethical guarantees and requirements for experimentation on human beings and animals and the requirements established in Spanish legislation in the field of biomedical research, protection of personal data, and bioethics, and adhered to the fundamental principles of the Declaration of Helsinki and the European Convention on Human Rights. Informed consent was obtained from the responsible tutors of the players.

## Results

The aim of this investigation is to assess the impact of an attention intervention among soccer players, using a video game. To assess the video game’s impact on the athletic performance of these players, an analysis of various variables was conducted, categorized into 3 main groups: the first group focused on the video game itself, examining the success rate in the game, EMG activity, and EDA; the second group encompassed decision-making variables related to the athletic performance component in soccer players; and in the third group, a subjective evaluation of each soccer player’s level of attention was obtained from their coach.

Regarding the variables analyzed in the video game, a significant improvement in the success rate of the soccer task was observed in the EG following the intervention program, as indicated by the level achieved. Both groups exhibited no differences in terms of success rate before undergoing training using the video game. However, after the 6-week duration, the EG demonstrated a substantial increase in the obtained score compared to the CG (Table 3).

Our findings indicate that there were no significant differences in EMG activity and assessed sweat between the 2 groups (Table 4).

**Figure 3.**
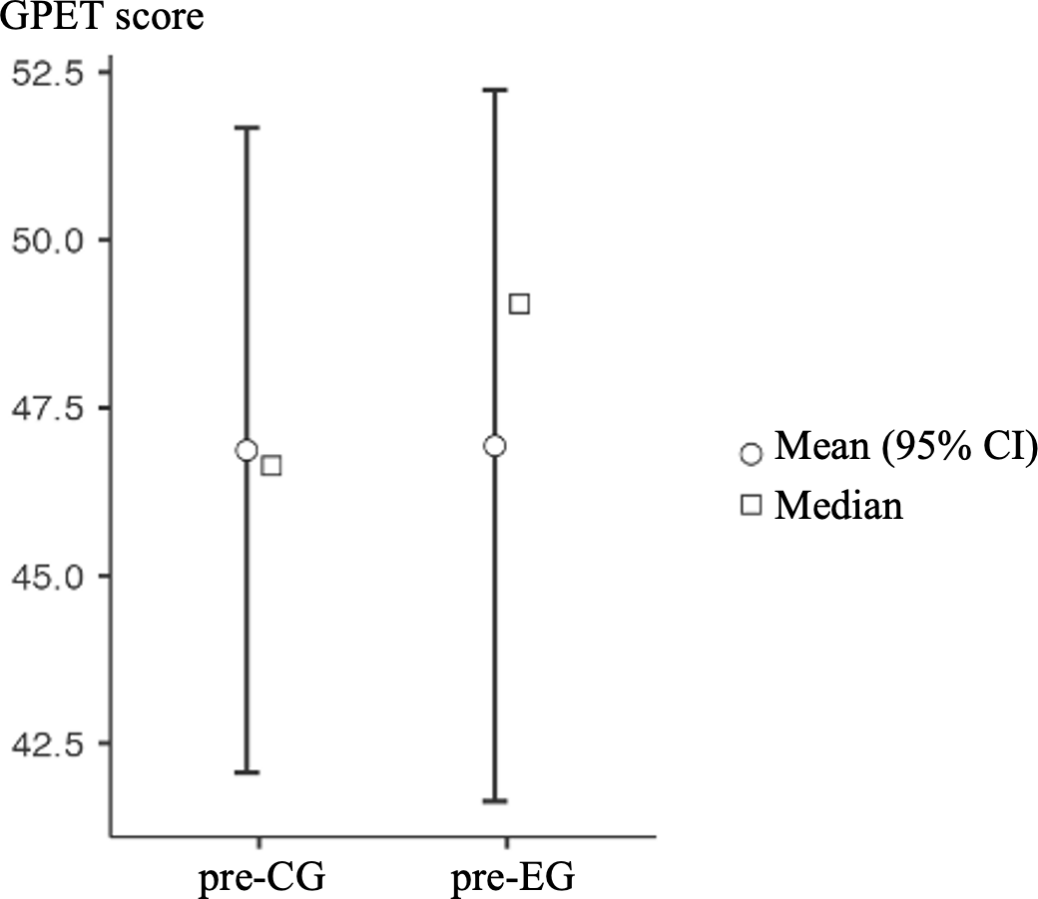
Comparison between the groups before the training period. CG: control group; EG: experimental group; GPET: Game Performance Evaluation Tool.

Regarding the set of variables assessed using the GPET tool, both groups underwent evaluation before and after the implementation of the attention training program using the video game. The variables were categorized as successful or failed actions based on the prevailing decision or execution mechanism, and they were further classified as attacking or defensive actions. All data were analyzed and are presented as percentages. Furthermore, the change in percentages for each variable was calculated by subtracting the values to determine both inter- and intragroup differences, and these differences were evaluated. See Figures3  4.

Regarding the mean score, a significant difference was observed between the EG and CG after the intervention period (pre-CG mean 46.2, SD 12.5; pre-EG mean 46.9, SD 13.8; *P*=.95; Cohen *d*=−0.01; post-CG mean 40.7, SD 13.7; post-EG mean 59.7, SD 17.3; *P*=.01; Cohen *d*=−1.73). As indicated, before the application of the attention training program using the video game, both groups showed relatively similar variables in which the decision mechanism predominated, as well as in those in which the execution mechanism predominated, the differences between both groups being less than 10%. Only 2 decision mechanism variables had values above 10% but never exceeding 15% (Shot_D=13/100, 13%; Marking_with_Ball_D=12/100, −12%).

Regarding the EG, following a 6-week intervention, all variables underwent some degree of modification, with minor changes below 15%. The variables that experienced the most substantial changes were Fixing_D (27/100, −27%), Fixing_E (37/100, −37%), Defensive_blocking_D (28/100, −28%), Marking_with_Ball_D (22/100, −22%), and Interception_D (18/100, −18%). The minus sign in these changes indicates that the percentage for each variable decreased after the intervention period. In the case of the CG, 9 variables showed changes exceeding 15%, with 5 of them surpassing 30%. On comparing the percentage changes between the 2 groups after the intervention, all variables had higher values in the EG than in the CG. The only variable that had higher values in the CG was the shot, on evaluation of both the decision mechanism (Shot_D=−1.43) and the execution mechanism (Shot_E=−1.69). However, the differences observed in both cases did not exceed 2 units.

**Table 3.** Comparison of success in the video game between groups before and after the intervention.

Variable	Before^a^		After^b^	
	CG^c^	EG^d^	CG	EG
Success, mean (SD)	1.66 (0.51)	1.5 (0.54)	1.83 (0.40)	4.16 (0.75)

a *P*=.61.

b *P*<.001.

c GC: control group.

d EG: experimental group.

**Table 4.** Comparison of EMG^a^ activity and EDA^b^ between groups before and after the intervention.

Variable	Before			After		
	CG^c^	EG^d^	*P* value^e^	CG	EG	*P* value
EMG activity (µV), RMS^f^ (SD)	1.66 (0.10)	1.71 (0.09)	.50	1.78 (0.12)	1.72 (0.05)	.21
EDA (µΩ), RMS, (SD)	25.11 (0.07)	25.81 (0.88)	.08	25.51 (0.48)	25.01 (0.85)	.20

a EMG: electromyographic.

b EDA: electrodermal activity.

c CG: control group.

d EG: experimental group.

e *P*≤.05 was considered significant.

f RMS: root-mean-square.

**Figure 4.**
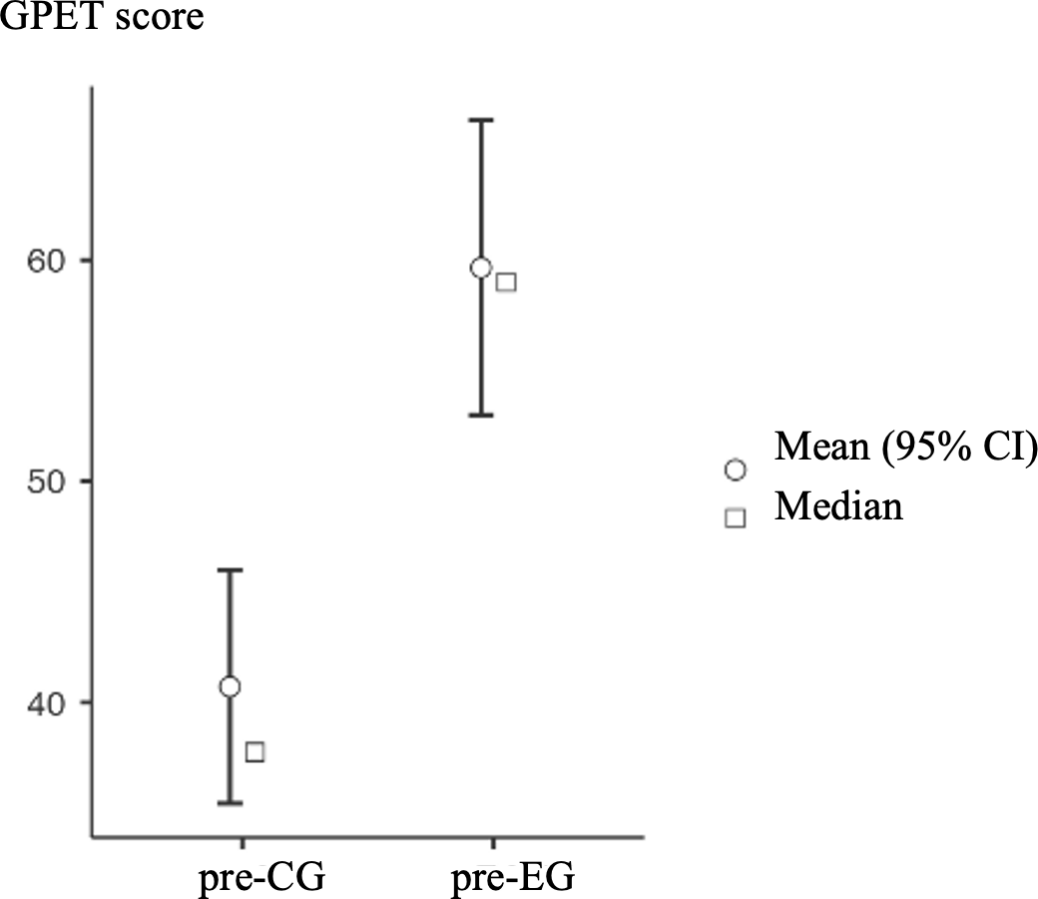
Comparison between groups after the training period. CG: control group; EG: experimental group; GPET: Game Performance Evaluation Tool.

Furthermore, it is evident that in the set of variables related to attack actions, the changes are more pronounced in variables associated with the execution mechanism than with those associated with the decision mechanism, except for dribbling and fixing. Conversely, in the set of variables related to defensive actions, the changes were more significant in the decision variables than in the execution variables, except for the variables of marking with the ball and marking without the ball.

Finally, the results obtained from the interview-based subjective evaluation of the soccer players’ attention levels are presented in Table 5.

**Table 5.** Subjective levels of attention evaluated by the coach.

Variable	Control group, mean (SD)	Experimental group, mean (SD)	*P* value^a^
Subjective level of attention	4.83 (1.47)	5.83 (1.16)	.22

a *P*≤.05 was considered significant.

## Discussion

### Principal Findings

The primary aim of this study was to develop a video game targeted at enhancing the attentional skills of young soccer players. By providing coaches with a tool for training the psychological aspect of attention, positive effects on various performance variables in soccer players could be observed. Following the intervention program, the EG demonstrated improvements in video game performance, indicating significant progress in overcoming the challenges presented by the game. These findings are consistent with those of a previous study [23] that reported improvements in attention execution through software interventions and considered using video games as an interesting tool for improve cognitive variables [35] in this population [36]. While our study did not use a specific attention assessment such as the D2 Attention Test [37] used by Reigal et al [23]x, one of our key findings is that alongside attention training through the video game, we evaluated decision-making related to soccer performance.

To monitor somatic factors such as muscle activity and skin conductance, we used EMG activity and EDA measurements for all participants. Research suggests that cognitive control and management of the somatic aspects of anxiety could impact attention levels [38]. In our study, we specifically collected EMG activity data from the hand extensor muscles. Our evaluation methodology aligned with that developed by Palkowski and Redlarski [39], who assessed EMG activity during various hand gestures. Our results revealed similar EMG activity patterns during hand opening actions, comparable to the postures observed when our sample participants placed their nondominant hand on the table while using the dominant hand to operate the computer mouse.

However, our analysis of EDA did not reveal any significant differences between the groups. This lack of divergence in the somatic response to anxiety suggests potential heightened control over the outward display of anxiety during the test. Surprisingly, both the CG and the EG obtained similar results. One possible explanation for this finding could be the presence of the play component, as both EMG activity and EDA evaluations were conducted during the practice of the video game. These results are aligned with the findings of Pop-Jordanova and Pop-Jordanov [40], who observed similar skin resistance outcomes when assessing individuals using biofeedback in static and calm situations. However, it appears that the video game may not be an effective tool for regulating somatic anxiety.

The evaluation of performance-related variables in soccer players with the GPET confirms the perceptual improvements in decision-making and execution observed in the EG after engaging in the intervention program with the video game. This suggests that enhancing attention through video game practice has a positive impact on decision-making in relation to soccer performance. These findings are aligned with those of González-Víllora et al [41], who assessed decision-making and execution in soccer situations using the same tool and observed significant differences between groups. Thus, our results further support the notion that the video game can serve as a valuable tool for enhancing the psychological aspect of attention in young soccer players, yielding positive effects on various performance variables in soccer.

Regarding shooting decisions, the CG demonstrated higher results than the EG. One possible explanation for this is that the EG developed attentional skills through the video game intervention, leading to improved decision-making during soccer and consequently reducing the frequency of shooting attempts. While no significant differences were observed between the 2 groups in the coach’s subjective evaluation of their players’ attentional mechanisms, it is evident that the video game serves as an effective tool for decision-making training. Moreover, it shows promise as a tool for detecting attentional impairments or abnormalities, highlighting its potential beyond its training benefits.

### Limitations

Despite our study reporting that this video game shows promise in improving attention in young soccer players, there are clear limitations that should be explained. Among the most important limitations of this study is that the sample size was small. This study comprised 12 soccer players, all members of a youth team of a first-level soccer team of the Spanish League. Although they comprised 100% of the team and effectively represented that team, our findings cannot be generalized; hence, it would be interesting to increase the sample size to verify our data.

The duration of the intervention was 6 weeks. Future studies could evaluate the effect of the intervention over a greater period, verifying if the effect on the improvement of some attentional processes assessed using the GPET was positive. In addition, the timing of the season where this attentional training program was integrated through the video game may also have been a limitation. Future studies could evaluate what happens at different times of the season.

Furthermore, somatic variables such as EDA and EMG activity were evaluated. Undoubtedly, the complexity underlying their evaluation is an important limitation in terms of reproducibility. Nevertheless, these variables were analyzed with the aim of determining whether there were any somatic issues that prevented the soccer players from being able to play the video game and obtain the best possible results.

Finally, there is a contextual limitation regarding the analysis of attention in real soccer situations. Although the ecological nature of the GPET has been proven, it is still a test that evaluates only the attention mechanism and the decision mechanism in soccer situations. This limitation could be overcome if future studies investigate what happens during a real match after attention training using the video game. Nevertheless, this evaluation was performed using the GPET to standardize the procedure so that all participants were evaluated in the same way and under the same conditions.

## Supplementary material

10.2196/52275 Checklist 1 CONSORT-EHEALTH (Consolidated Standards of Reporting Trials of Electronic and Mobile Health Applications and Online Telehealth) checklist (V 1.6.1).

## Abbreviations

CG: control group
EDA: electrodermal activity
EG: experimental group
EMG: electromyographic
GPET: Game Performance Evaluation Tool
